# Loss of *wbpL* disrupts *O*‐polysaccharide synthesis and impairs virulence of plant‐associated *Pseudomonas* strains

**DOI:** 10.1111/mpp.12864

**Published:** 2019-09-27

**Authors:** Alexander Kutschera, Ursula Schombel, Michelle Wröbel, Nicolas Gisch, Stefanie Ranf

**Affiliations:** ^1^ Technical University of Munich Phytopathology, TUM School of Life Sciences Weihenstephan 85354 Freising‐Weihenstephan Germany; ^2^ Research Center Borstel, Leibniz Lung Center Division of Bioanalytical Chemistry, Priority Area Infections Parkallee 1‐40 23845 Borstel Germany

**Keywords:** lipopolysaccharide, motility, *O*‐polysaccharide, *Pseudomonas syringae*, virulence

## Abstract

Despite its importance for membrane stability and pathogenicity of mammalian pathogens, functions of the *O*‐polysaccharide (OPS) of lipopolysaccharide (LPS) remain unclear in plant‐associated bacteria. Genetic information about OPS biosynthesis in these bacteria is largely missing. Genome analysis of various plant‐associated *Pseudomonas* strains revealed that one of the two known OPS biosynthesis clusters from *Pseudomonas aeruginosa* PAO1, the common polysaccharide antigen (CPA) gene cluster, is only conserved in some strains of the *Pseudomonas fluorescens* group. For the *O*‐specific antigen (OSA) biosynthesis cluster, the putative genomic position could be identified, but orthologues of most functional important OSA biosynthesis enzymes could not be detected. Nevertheless, orthologues of the glycosyltransferase WbpL, required for initiation of CPA and OSA synthesis in *P. aeruginosa* PAO1, could be identified in the analysed *Pseudomonas* genomes. Knockout mutations of *wbpL* orthologues in *Pseudomonas syringae* pv. *tomato* DC3000 (*Pst*) and *Pseudomonas cichorii* ATCC10857/DSM50259 (*Pci*) resulted in strains lacking the OPS. Infection experiments of *Arabidopsis thaliana* plants revealed a reduced entry into the leaf apoplast after spray inoculation and a reduced apoplastic amplification of *Pst* ∆*wbpL*. Stab and spray inoculation of lettuce (*Lactuca sativa*) leaves with *Pci* ∆*wbpL* causes reduced infection symptoms compared to the wild‐type strain. Furthermore, swarming motility was reduced in ∆*wbpL* mutants of *Pst* and *Pci*. This might be a possible reason for reduced bacterial titres after surface inoculation and reduced bacterial amplification in the plant. Our results imply that the presence of lipopolysaccharide OPS is required for efficient host colonization and full virulence of plant‐pathogenic *Pseudomonas* bacteria.

## Introduction

The genus *Pseudomonas* comprises several plant‐associated bacteria, including versatile plant pathogens that infect crops and cause substantial economic damage (Vanneste, 2017; Young, 2010). For successful infection, bacteria have to overcome various chemical and physiological barriers and induced defence responses that depend on the plant species and their habitat. Therefore, bacteria rely on various defence characteristics themselves.

In Gram‐negative bacteria, the outer membrane (OM) is crucial for bacterial survival. It confers structural stability to bacterial cells and, as a restrictive permeability barrier, protects bacteria from antimicrobial substances while facilitating material exchange with the environment (Alexander and Rietschel, 2001). Lipopolysaccharide (LPS) is an essential OM component and takes part in interactions between bacterial cells and their environment. It can mask the bacterial cell surface to avoid opsonization and possesses endotoxic activity in mammals (Lukácová *et al.*, 2008; Needham and Trent, 2013; Trent *et al.*, 2006).

LPS consists of three covalently linked domains with different chemical and biological properties: the lipophilic lipid A (LA) moiety, the hydrophilic oligosaccharide core region (core‐OS) and *O*‐polysaccharide (OPS) (Alexander and Rietschel, 2001). Commonly, the OPS is a heteropolymeric *O*‐specific antigen (OSA) that has a highly diverse composition among bacterial species and strains, and determines their serological and antigenic specificity. In *Pseudomonas aeruginosa*, the common polysaccharide antigen (CPA) can also be found instead of, or in parallel with, the OSA (Raetz and Whitfield, 2002). The length of the OPS chain ranges from one to >50 repeats and influences cell surface hydrophobicity. LPS molecules of different OPS chain length can occur in parallel to OPS‐deficient variants on a single bacterial cell (Alexander and Rietschel, 2001). OPS protects the bacterium from unfavourable environments by providing a steric as well as a diffusion barrier, e.g. to antibacterial agents that target the interior core and LA parts of LPS (Ranf, 2016). Due to its outermost localization and hydrophilic properties, OPS mediates adhesion to host surfaces and possibly host selectivity (Bogino *et al.*, 2013). Through adherence to other bacteria, it is involved in formation of biofilms, which likely is the preferred growth mode in host tissues (Bogino *et al.*, 2013; Dongari‐Bagtzoglou, 2008). Bacteria lacking OPS have a reduced viability and cannot persist under stress conditions, such as in a host (Raetz and Whitfield, 2002). Additionally, distribution of OPS chain length is species‐specific (King *et al.*, 2014; Murray *et al.*, 2006; Rahman *et al.*, 1997; Tran *et al.*, 2014; Van den Bosch *et al*., 1997). Variances in the OPS structure are commonly linked to an altered pathogenicity (Lerouge and Vanderleyden, 2002; Lukácová *et al.*, 2008).

In *P. aeruginosa*, two gene clusters are known to code for enzymes that are required for the complete synthesis of the respective OPS type (Lam *et al.*, 2011). The synthesis of the homopolymeric CPA is ABC‐transporter‐dependent and mainly takes place at the cytoplasmic face of the inner membrane (IM). The polysaccharide is built up on an undecaprenyl pyrophosphate (Und‐PP)‐carrier through addition of saccharide residues to the non‐reducing terminus by glycosyltransferases. An ABC‐transporter system transfers the fully synthesized polysaccharide to the periplasmic face of the IM (Greenfield and Whitfield, 2012). *P. aeruginosa* OSA is synthesized via the Wzy‐dependent pathway. Single OSA repeating units are assembled on an Und‐PP‐carrier at the cytoplasmic face of the IM and transported to the periplasmic face by the flippase Wzx. In the periplasm, the saccharide subunits are joined with the nascent OSA at its reducing terminus by the polysaccharide polymerase Wzy. The OSA chain length is regulated by the polysaccharide copolymerase Wzz (Islam and Lam, 2014). Both pathways share the initiation step, the addition of a first monosaccharide to the Und‐PP‐carrier by the glycosyltransferase WbpL (Lam *et al.*, 2011). In the periplasm, the completed OPS is transferred from the Und‐PP‐carrier to the LA‐core‐OS unit by the OPS ligase WaaL (Whitfield *et al.*, 1997).

LPS mutants from several members of the *Rhizobiales* lacking an OPS are defective in symbiosis because of premature abortion of infection threads (Carlson *et al.*, 2010; Lerouge and Vanderleyden, 2002; Ormeño‐Orrillo *et al.*, 2008). This suggest that OPS plays a role in root infection during symbiotic plant‐bacteria interactions. Similar to mammalian pathogens, the protective nature of OPS is considered a key factor for bacterial survival and growth in plants. Since resistance against hostile environmental conditions is a premise for a successful infection, it is assumed that OPS is essential for plant‐pathogen virulence (Kutschera and Ranf, 2019a). Several studies with OPS‐defective mutants of *Erwinia* spp., *Ralstonia solanacearum*, *Xanthomonas axonopodis* pv. *citri* and *Xylella fastidiosa* showed a loss or strong reduction of virulence in the host plant (Berry *et al.*, 2009; Drigues *et al.*, 1985; Li *et al.*, 2014; Petrocelli *et al.*, 2012; Rapicavoli *et al.*, 2018; Schoonejans *et al.*, 1987). Rudolph *et al. *(1989) reported a strongly reduced colonization of the leaf mesophyll and a decreased virulence of OPS‐deficient *Pseudomonas syringae* pv. *phaseolicola*. They postulated that OPS confers compatibility with the plant and determines host specificity (Rudolph, 2001). Although the molecular OPS composition of many plant‐associated bacteria has been elucidated in recent years, the genetic background of OPS biosynthesis remains unknown in many plant‐associated *Pseudomonas* strains. Differences in OPS chain length distribution between different pathovars/strains raise the question about the regulatory mechanisms determining the prevalence for specific OPS sizes analogous to other bacterial species (Kalynych *et al.*, 2011; King *et al.*, 2014; Kutschera and Ranf, 2019b; Whitfield *et al.*, 1997).

In this work, we shed light on the genetics of OPS synthesis in *P. syringae* and related plant‐associated bacteria through genome and proteome sequence analysis. We identified the glycosyltransferase WbpL, which is conserved in all examined strains, as a key component of OPS synthesis. However, we could not identify a complete OPS gene cluster responsible for OPS synthesis in *P. syringae* and related species. Deletions of the identified *wbpL* orthologues in *P. syringae* pv. *tomato* DC3000 (*Pst*) and *Pseudomonas cichorii* ATCC10857/DSM50259 (*Pci*) resulted in OPS‐deficient mutants. Infection of *Arabidopsis thaliana* with *Pst* ∆*wbpL* or lettuce (*Lactuca sativa*) leaves with *Pci* ∆*wbpL* was delayed compared to the wild‐type strains, but neither a rapid decline of bacterial titres nor a complete avirulence of the ∆*wbpL* mutants was observed. Finally, our findings suggest that OPS influences motility and thereby affects colonization of plant hosts.

## Results

### Partial conservation of OPS‐synthesis gene clusters in phytopathogens

We conducted comparative analyses with predicted protein sequences from the genomes of various *Pseudomonas* species to identify LPS biosynthesis components and to reveal similarities and differences in OPS synthesis. Most *P. aeruginosa* strains produce two different OPS types, OSA and CPA. The corresponding genes are organized in two independent gene clusters (Hao *et al.*, 2013; Samuel and Reeves, 2003). Putative orthologues of the proteins encoded by these OPS gene clusters were identified in the investigated predicted proteomes, followed by synteny analysis of the respective genes in the genome context.

**Figure 1:**
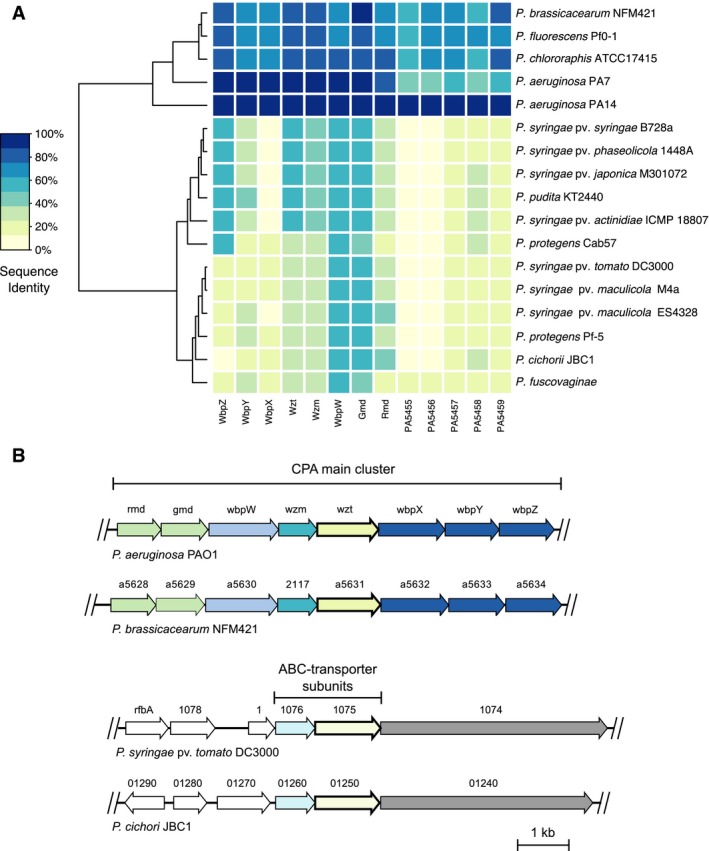
Comparative analysis of the common polysaccharide antigen (CPA) cluster. The CPA synthesis gene cluster could not be identified in most of the analysed *Pseudomonas* genomes. (A) Heatmap of results from a BLASTP search for homologous CPA biosynthesis enzymes in different predicted proteomes. *P. aeruginosa* PAO1 sequences were used as reference (e‐value cut‐off of 10^−9^). Phylogenetic tree shows the Euclidean distance calculated from the BLASTP identities. (B) Gene structure of the *P. aeruginosa* PAO1 and *P. brassicacearum* NFM421 CPA cluster and locus of putative ABC‐transporter subunit homologues in *P. syringae* pv. *tomato* DC3000 and *P. cichorii* JBC1. Colour indicates the respective gene hits in the genomes.

The main CPA cluster is highly conserved among *P. aeruginosa* strains and consists of eight genes (*rmd*–*wbpZ*, Fig. 1). Additionally, a proximal cluster of five genes (*PA5455*–*PA5459*) is required for CPA synthesis in *P. aeruginosa* PAO1 and PA14 (Lam *et al.*, 2011). BLASTP search for CPA biosynthesis enzymes, using *P. aeruginosa* PAO1 sequences as query, revealed that some homologues are present in the respective proteomes (Fig. 1). Sequence identities of most BLASTP hits were below 50%, whereas hits with over 98% sequence identity for all query sequences were retrieved in the *P. aeruginosa* PA14 proteome. For *P. aeruginosa* PA7, sequence identities of the respective hits ranged from 82.8% to 98.5% (main cluster) and from 41.3% to 54.7% (additional cluster). Likewise, *P. fluorescens* Pf0‐1, *P. chlororaphis* ATCC17415 and *P. brassicacearum* NFM421 showed sequence identities ranging from 51.0% to 88.2% for all query sequences. As previously reported (Lam *et al.*, 2011; Silby *et al.*, 2009), *P. aeruginosa* serovars, *P. fluorescens* Pf0‐1, *P. chlororaphis* ATCC17415 and *P. brassicacearum* NFM421 share a common organization of CPA synthesis genes (Fig. 1). No similar gene organization was observed in the remaining analysed genomes. Most of the coding sequences of the putative CPA synthesis enzyme homologues are distributed in the respective genome and a few occur in pairs. For example, genes corresponding to the homologues of the two ABC‐transporter subunits Wzt (PA5450) and Wzm (PA5451) of *Pst* and *Pci* JBC1 are located next to each other in the respective genomes. The location of these genes is similar in *Pst* and *Pci* JBC1, but the synteny in the context of the *P. aeruginosa* PAO1 CPA gene cluster is not conserved (Fig. 1B). According to the low sequence identities of the putative homologues, these genes likely code for ABC‐transporter subunits not involved in CPA biosynthesis. Except for the *P. aeruginosa* strains, *P. fluorescens* Pf0‐1, *P. chlororaphis* ATCC17415 and *P. brassicacearum* NFM421, the overall results of the comparative analysis indicate that the CPA cluster is not conserved in all plant‐associated *Pseudomonas* species. In summary, the analyses suggest an OPS synthesis mediated by enzymes not orthologous to the CPA biosynthesis in *P. aeruginosa* PAO1.

**Figure 2:**
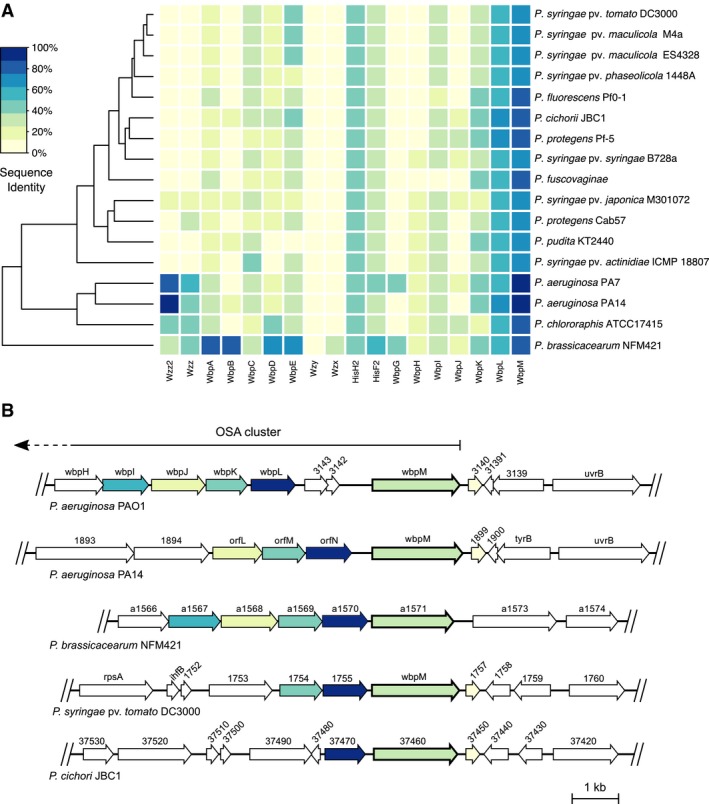
Comparative analysis of the *O*‐specific antigen (OSA) cluster. The putative locus and homologues of genes from the OSA synthesis cluster could be identified in *Pseudomonas* spp. (A) Heatmap of results from a BLASTP search for homologous OSA biosynthesis enzymes in different predicted proteomes. *P. aeruginosa* PAO1 sequences were used as reference (e‐value cut‐off = 10^−9^). Phylogenetic tree shows the Euclidean distance calculated from the BLASTP identities. (B) Gene structure of the upstream part of the *P. aeruginosa* PAO1 OSA cluster and hits from synteny analysis of corresponding genes in other *Pseudomonas* species. Colour indicates the respective gene hits in the genomes.

The OSA biosynthesis cluster is not as highly conserved as the CPA cluster due to the intrinsic variation of OSA structures of the *P. aeruginosa* serovars. While the cluster content differs considerably in serovar‐specific glycosyltransferases, the locus of the gene cluster and regulatory enzymes of OSA synthesis such as the chain length regulator Wzz are conserved. The OSA cluster position is defined by the proposed border genes *wzz* (PA3160) and *wbpM* (PA3141, saccharide epimerase/dehydratase). A comparative analysis of *P. aeruginosa* PA14 and *P. aeruginosa* PA7 OSA‐biosynthesis enzymes confirms these variations (Raymond *et al.*, 2002) (Fig. 2). In other *Pseudomonas* genomes, orthologues of the border gene *wbpM* with amino acid sequence identities ranging from 68.6% to 77.0% of the corresponding predicted proteins were found in a similar gene context. In all *Pseudomonas* strains, orthologues of the glycosyltransferase WbpL with sequence identities ranging from 59.2% to 66.0% were identified. Sequence identities of putative WbpK orthologues were considerably lower (36.2–42.4%). In *P. aeruginosa* PAO1, the corresponding genes (*wbpL*: PA3145, *wbpK*: PA3146) are located upstream of *wbpM*. Synteny studies revealed a conserved location of the respective *wbpL* orthologues in all analysed *Pseudomonas* strains, whereas location of *wbpK* is only conserved in a few strains (Fig. 2B). For other proteins encoded by the *P. aeruginosa* PAO1 OSA cluster, the analysis of most proteomes yielded no or only low sequence identity hits that do not seem to be located in a similar OSA cluster context. Notably, genomes of *P. chlororaphis* ATCC17415 and *P. brassicacearum* NFM421 contain putative orthologues of *wzz*. Analysis of the *P. brassicacearum* NFM421 genome revealed several other putative gene orthologues of the *P. aeruginosa* PAO1 OSA cluster with a conserved synteny (Fig. 2B).

Overall, only two to three of 17 genes in the *P. aeruginosa* PAO1 OSA cluster appear to be conserved in sequence and position in the majority of the analysed *Pseudomonas* strains (Fig. 2B). Hence, this short cluster might not be sufficient for the synthesis of a complete OPS in these strains. Nevertheless, the high sequence identities of the putative WbpM and WbpL orthologues suggest that these proteins function in OPS synthesis. In *P. aeruginosa*, WbpL initiates OSA as well as CPA synthesis (Bélanger *et al.*, 1999; Rocchetta *et al.*, 1998, 1999). The putative WbpL orthologues of the analysed *Pseudomonas* strains likely also initiate OPS synthesis.

### Knockout of *wbpL* results in disruption of OPS synthesis

**Figure 3:**
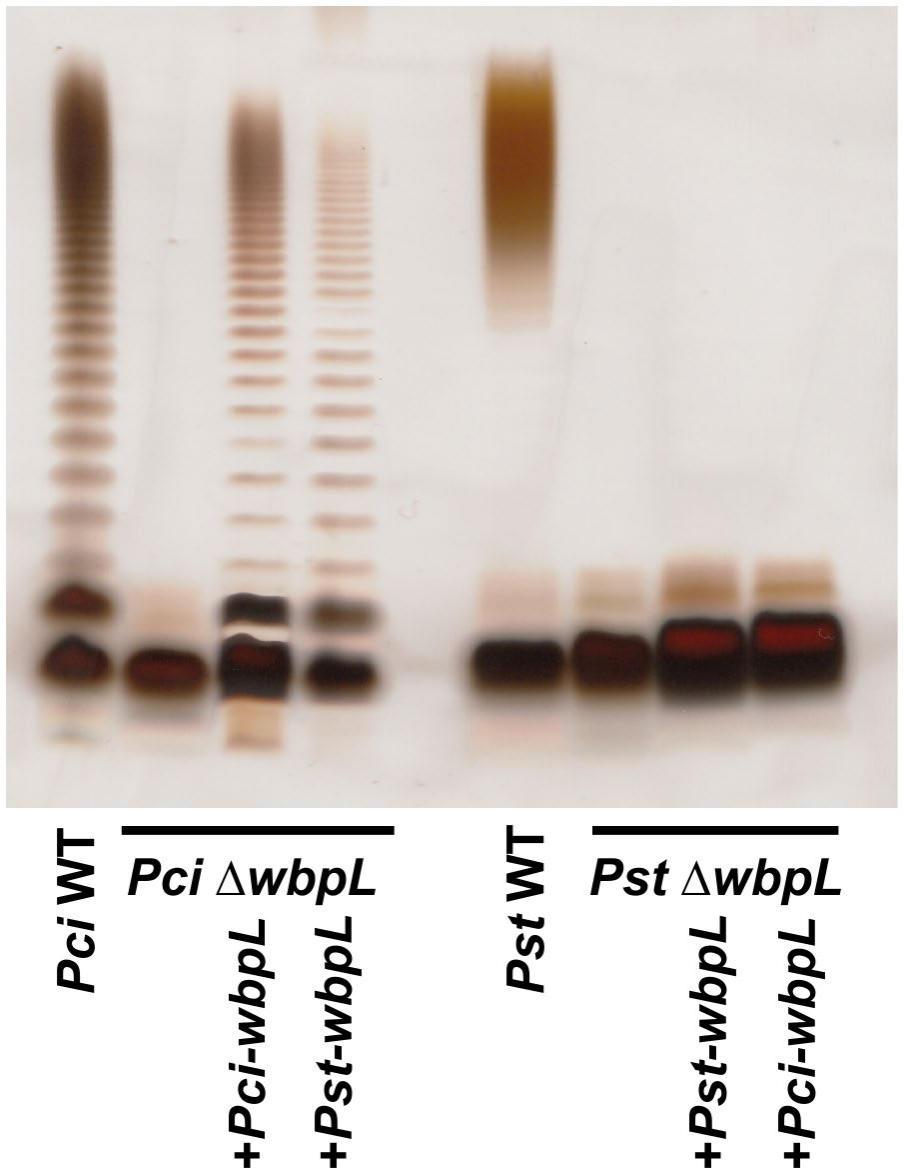
12% Bis‐Tris NuPAGE gel and silver staining of lipopolysaccharide (LPS) preparations from *Pseudomonas cichorii* ATCC10857/DSM50259 (*Pci*) and *Pseudomonas syringae* pv. *tomato* DC3000 (*Pst*) wild‐type (WT), ∆*wbpL::Gm^R^* (∆*wbpL*) deletion mutant strains and mutant strains complemented with plasmid‐expressed *wbpL* orthologues from *Pci* or *Pst*. LPS was isolated by hot phenol‐water extraction followed by a phenol‐chloroform‐petroleum ether extraction. 2 μg of ∆*wbpL* LPS, for all other LPS 4 μg were applied per lane.

Knockout of *wbpL* in *P. aeruginosa* disrupts OPS synthesis (Bélanger *et al.*, 1999). Knockout of the putative *wbpL* genes in *Pst* and *Pci* should reveal if the corresponding proteins are also involved in OPS synthesis. We deleted the identified *wbpL* candidates in *Pci* (PCH70_40470 in *P. cichorii* JBC1) and *Pst* (PSPTO_1755) by replacement of the *wbpL* gene sequence with a gentamicin resistance cassette (Gm^R^). Three independent transformants of each strain were analysed for alterations in OPS biosynthesis. Silver‐stained SDS‐PAGE of crude LPS preparations indicated a loss of OPS synthesis. The ∆*wbpL::Gm^R^* (∆*wbpL*) deletion mutants showed no noticeable growth phenotypes compared to the wild‐type strains. For further analysis, LPS was isolated by hot phenol/water and phenol/chloroform/petroleum ether‐extraction from one selected transformant per strain. Bis‐Tris NuPAGE/silver staining of these LPS preparations (Fig. 3) confirmed that LPS of the *Pci* and *Pst* ∆*wbpL *mutants lacks OPS.

### Complementation of the gene knockout with *Pci‐wbpL* or *Pst‐wbpL* partially restores OPS synthesis in *Pci* ∆*wbpL*

Complementation strains of *Pst* ∆*wbpL* and *Pci* ∆*wbpL *were generated to exclude polar effects of the Gm^R^ insertion. Complementation plasmids containing the *wbpL* orthologue from *Pci* (PCH70_40470 in *P. cichorii* JBC1) or *Pst* (PSPTO_1755) under control of the Gm^R^ promoter were transferred into the respective ∆*wbpL* mutant strain. SDS‐PAGE analysis of crude LPS preparations from two transformants per mutant strain indicated successful reconstitution of OPS biosynthesis in the complemented *Pci* ∆*wbpL* but not in *Pst* ∆*wbpL *strains. LPS isolation by hot phenol/water and phenol/chloroform/petroleum ether‐extraction and Bis‐Tris NuPAGE/silver staining confirmed the recovery of OPS biosynthesis in *Pci* ∆*wbpL *strains containing the *Pci‐wbpL* expression plasmid (Fig. 3). However, LPS preparation of *Pst* ∆*wbpL *strain complemented with the *Pst‐wbpL* gene did not show reconstitution of OPS synthesis. To assess whether the *Pst‐wbpL* complementation plasmid is functional and transient expression of the putative WbpL orthologues in *Pst* is sufficient to complement the LPS phenotype, *Pci* ∆*wbpL *was transformed with the *Pst‐wbpL* complementation plasmid and *Pst* ∆*wbpL *with the *Pci‐wbpL* complementation plasmid (cross‐complementation). SDS‐PAGE and silver staining of crude LPS preparations from two transformants each suggested that the cross‐complemented *Pci* ∆*wbpL *but not *Pst* ∆*wbpL *strains showed a recovery of OPS biosynthesis. Analysis of LPS isolated by hot phenol/water and phenol/chloroform/petroleum ether‐extraction and Bis‐Tris NuPAGE/silver‐staining confirmed this result (Fig. 3). The presence of LPS substituted with OPS in *Pci* ∆*wbpL* cross‐complemented with *Pst‐wbpL* demonstrates that the *Pst‐wbpL* complementation plasmid is functional, and transient expression of the *Pst* WbpL orthologue principally complements the *Pci* ∆*wbpL *LPS phenotype, although apparently not to the full extent of the endogenous WbpL. LA and core‐OS‐OPS were isolated from *Pci* ∆*wbpL + Pci‐wbpL* by mild acid hydrolysis (Ranf *et al.*, 2015) and respective molecular structures were analysed and compared with wild‐type *Pci* LPS. No substantial structural alterations could be observed (as judged by mass spectrometry of core‐OS and ^1^H NMR of core‐OS with OPS; data not shown). Notably, hydrolysis of the LPS from the wild‐type strain resulted in a weight‐to‐weight ratio of 2:1 for the core‐OS versus core‐OS‐OPS, whereas for LPS from the complemented strain a ratio of 10:1 was observed. This was further corroborated by NuPAGE analysis using different LPS concentrations. A five‐times higher LPS concentration of the LPS isolated from the *Pci* ∆*wbpL* + *Pci‐wbpL *complementation strain compared to *Pci* wild‐type LPS shows a similar band pattern of OPS‐substituted LPS molecules with a simultaneous increase of staining intensity for OPS‐deficient LPS. A similar effect was observed for the LPS isolated from the *Pci* ∆*wbpL* + *Pst‐wbpL *complementation strain (Fig. S1). In summary, this indicates that the complementation was only partially successful as the complementation strains contain less LPS substituted with OPS.

**Figure 4:**
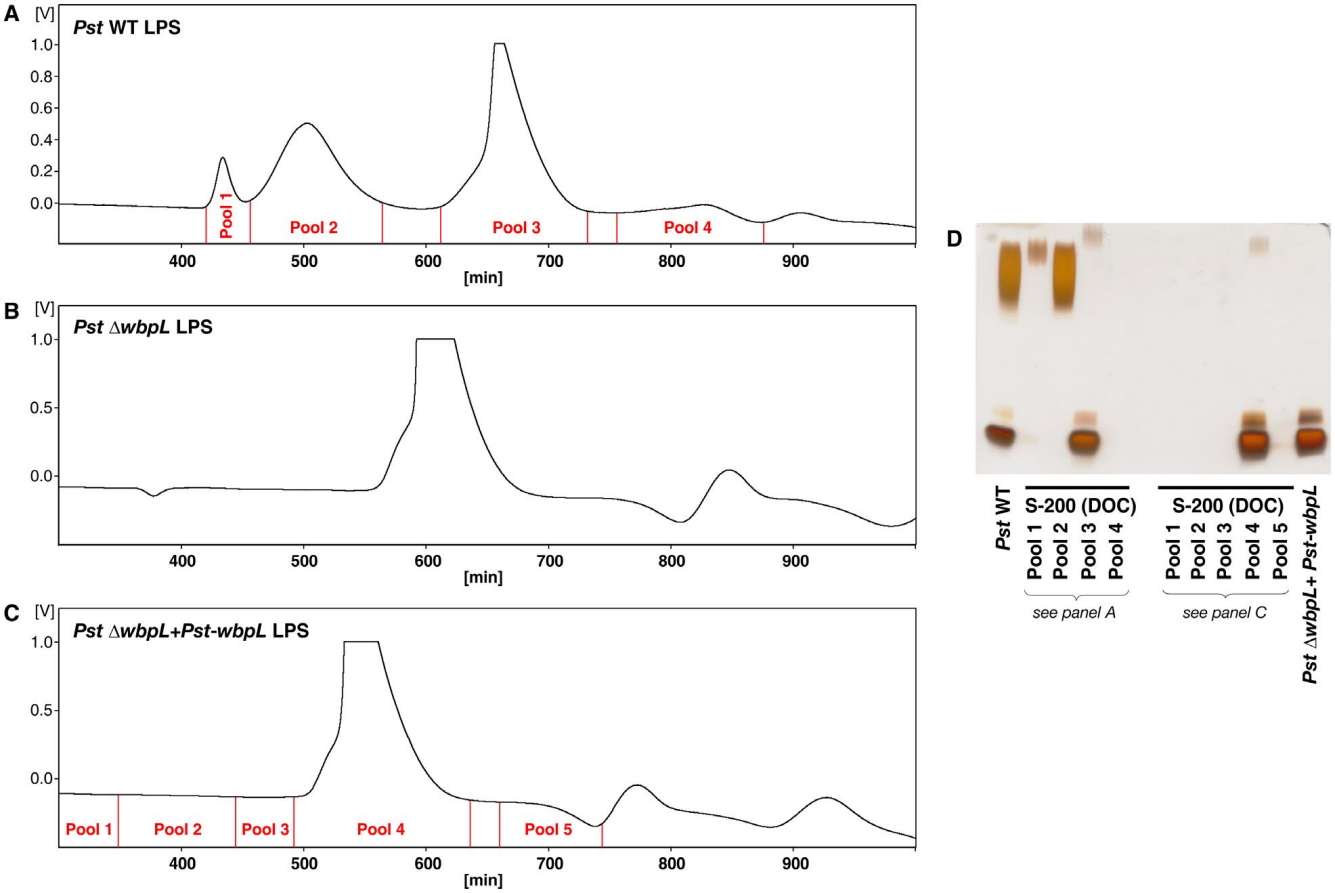
Fractionation of lipopolysaccharide (LPS) from *Pseudomonas syringae* pv. *tomato* DC3000 (*Pst*) (A) wild‐type (WT), (B) ∆*wbpL* and (C) complemented (∆*wbpL* + *Pst‐wbpL*) strain on Sephacryl S‐200 HR using a desoxycholate (DOC)‐containing buffer. The relevant regions of representative GPC chromatograms are depicted, including the chosen fractionation. In case of ∆*wbpL* + *Pst‐wbpL* strain, material of two comparable GPC runs were combined for further assays. (D) Silver‐stained 12% Bis‐Tris NuPAGE gel of the indicated combined column pools and the respective starting material of A and C. 4 μg of LPS were applied per lane.

To confirm the OPS‐loss in the *Pst* ∆*wbpL *complementation strain (+*Pst‐wbpL*) and to exclude inefficient staining as cause for the visual absence of signals in the high molecular weight region (as observed with crude extract of *Pst* (Kutschera and Ranf, 2019b), the LPS preparations of the *Pst* wild‐type, the ∆*wbpL* and the complemented ∆*wbpL* strain were fractionated by gel permeation chromatography (GPC) with a deoxycholate (DOC)‐containing buffer (Fig. 4A–C). NuPAGE analysis of LPS pools 1 and 2 from *Pst* showed signals in the high molecular weight region, while pool 3 contained OPS‐deficient LPS (Fig. 4D). GPC chromatograms and subsequent NuPAGE analysis of the ∆*wbpL* (Fig. 4B,D) and of the *Pst* ∆*wbpL *complementation strain (+*Pst‐wbpL*; Fig. 4C,D) prove the absence of OPS‐substituted LPS species. The sole presence of OPS‐deficient LPS in the complemented strain (pool 4 in Fig. 4C) shows that the OPS synthesis in *Pst* ∆*wbpL *was not reconstituted by transient *wbpL* expression.

### *Pst *∆*wbpL *and *Pci* ∆*wbpL* are less virulent than the wild‐type strains

**Figure 5:**
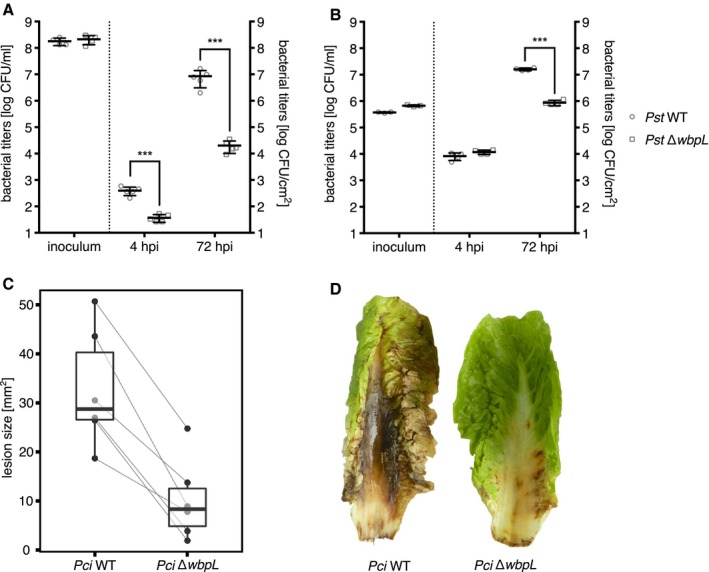
Infection experiments with *Pseudomonas syringae* pv. *tomato* DC3000 (*Pst*) and *P. cichorii* ATCC10857/DSM50259 (*Pci*) wild‐type (WT) and ∆*wbpL* deletion mutants. *Arabidopsis thaliana* plants were inoculated by spraying (A, *n* = 5, mean ± SEM) or syringe‐infiltration of leaves (B, *n* = 4, mean ± SEM) with *Pst* WT or *Pst* ∆*wbpL* suspension. Samples were taken 4 and 72 h post‐inoculation (hpi). (*P* < 0.001, multiple *t*‐test, experiments were repeated at least three times with similar results). (C) Lettuce midrib was stab infected with *Pci* WT and *Pci* ∆*wbpL* and the size of the emerging lesions was measured 2 days post‐inoculation (dpi) (Fig. S2A, *n* = 6, *P* < 0.05, Wilcoxon signed‐rank test, experiment was repeated at least three times with similar results). (D) Lettuce leaves were sprayed with *Pci* WT or *Pci* ∆*wbpL* suspensions and disease symptoms were monitored over time. The picture was taken 9 dpi. The original image is provided in the supporting information (Fig. S2B).

*Arabidopsis thaliana* plants were infected with *Pst* wild‐type and *Pst* ∆*wbpL* strains to evaluate effects of the WbpL knockout and the resulting OPS‐deficiency on virulence. Spray inoculation revealed a reduced bacterial titre in plants treated with *Pst* ∆*wbpL *compared to plants treated with wild‐type *Pst* (Fig. 5A). This difference had already been observed 4 h post‐inoculation (hpi). Bacterial titres were strongly increased at 72 hpi in both wild‐type and ∆*wbpL *mutant‐infected plants. The OPS‐deficient *Pst* ∆*wbpL *mutant, however, amplified to lower titres than the wild‐type strain. Spray inoculation mimics natural infection, since bacteria need to overcome stomatal immunity and actively enter the leaf interior. Thus, the initial differences in bacterial titres suggest an impaired capability of *Pst* ∆*wbpL *cells to enter the host tissue. To determine bacterial amplification in the leaf apoplast, *A. thaliana* leaves were pressure‐infiltrated with bacterial suspensions. Accordingly, wild‐type and mutant *Pst* bacterial titres were comparable at 4 hpi. At 72 hpi, *Pst* ∆*wbpL *titres were significantly lower than *Pst* wild‐type titres (Fig. 5B), suggesting that the apoplastic amplification rate of the mutant bacteria is reduced. Thus, the ability to enter the host as well as colonization of the apoplast is affected by the *wbpL* knockout and the concomitant OPS loss. Long‐term monitoring of *A. thaliana* inoculated with both *Pst* strains by pressure‐infiltration of leaves revealed that *Pst* ∆*wbpL* causes typical infection symptoms, but delayed compared to the wild‐type strain (Video S1). Stab inoculation of lettuce midrib with *Pci* ∆*wbpL* resulted in reduced lesion size compared to the wild‐type strain. Similarly, *Pci* ∆*wbpL* caused less severe disease symptoms when sprayed on lettuce leaves (Fig. 5C,D).

### WbpL knockout diminishes swarming motility

**Figure 6:**
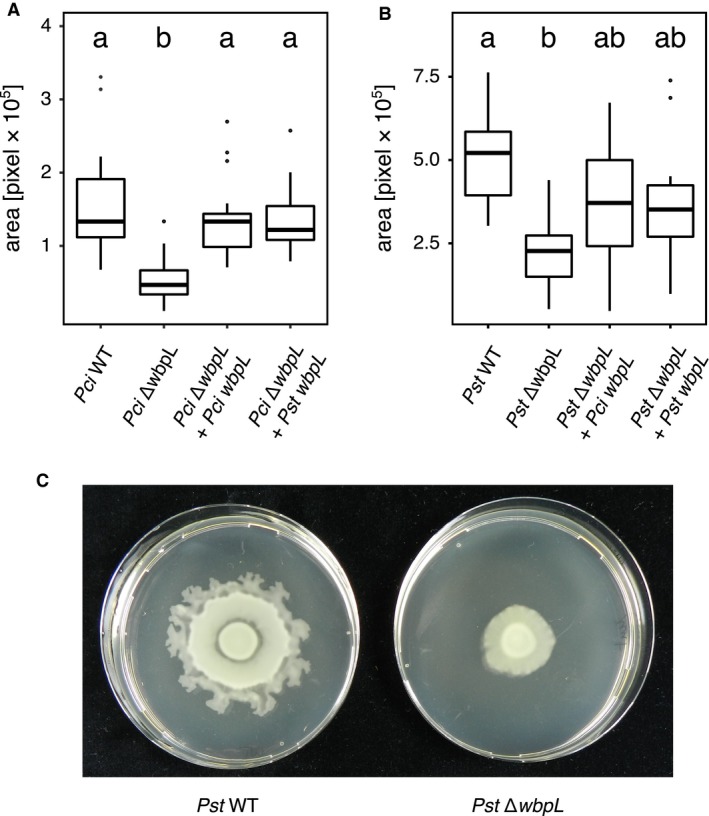
Comparison of swarming motility of *Pseudomonas syringae* pv. *tomato* DC3000 (*Pst* WT, A, *n* = 11, exemplary images in C) and *Pseudomonas cichorii ATCC10857/DSM50259* (*Pci* WT, B, *n* = 16) with the respective ∆*wbpL::Gm^R^* (∆*wbpL*) and *Pci* WbpL (*Pci*‐*wbpL*) or *Pst* WbpL (*Pst*‐*wbpL*) expressing complementation strains. Data from three independent experiments were pooled. Letters indicate statistical difference (*P* < 0.05) calculated by a Kruskal–Wallis rank sum test and post hoc pairwise comparisons using the Nemenyi‐test with chi‐squared approximation for independent samples.

The ability to move actively is crucial for the lifestyle of plant‐associated bacteria. In particular, swarming motility, collective and directional movements of bacteria on a surface, are important for plant colonization (Haefele and Lindow, 1987; Harshey, 2003; Zeng *et al.*, 2010). The swarming motility of *Pst* and *Pci* wild‐type and ∆*wbpL* strains was therefore determined *in vitro*. Swarming motility of ∆*wbpL *mutants was significantly reduced compared to the wild‐type in both strains (Fig. 6). For *Pci* ∆*wbpL *strains complemented with the respective native *Pci‐wbpL* and *Pst‐wbpL*, a significant increase of migration area could be observed compared to the mutant strain. In accordance with the lack of complementation of the LPS phenotype, transient expression of the *Pst* or *Pci wbpL* gene in *Pst* ∆*wbpL *did not reconstitute the motility phenotype. These results show that a loss of the OPS diminishes swarming motility in both bacterial strains.

## Discussion

LPS is a major virulence factor for many pathogenic Gram‐negative bacteria. OPS, a molecular substructure of LPS, has various well‐investigated virulence functions in mammalian hosts, such as complement resistance (Murray *et al.*, 2006) and influence on biofilm formation (Murphy *et al.*, 2014). However, little is known about OPS biosynthesis in plant‐associated bacteria and the role of OPS in plant infection. Here, we report that most of the analysed plant‐associated *Pseudomonas* strains lack orthologues of the *P. aeruginosa* CPA biosynthesis gene cluster. We identified a putative but short orthologue of the *P. aeruginosa* OSA biosynthesis gene cluster, which includes the conserved glycosyltransferase WbpL. By genetic knockout and complementation, we show that WbpL is essential for OPS synthesis in *Pst* and *Pci*. Infection assays of *A. thaliana* and lettuce with OPS‐deficient *Pst* or *Pci* strains, respectively, revealed a reduction but not a loss of virulence. Finally, our results indicate a possible link between loss of the OPS, reduced swarming motility and reduced virulence *in planta*.

### The genetic background of OPS synthesis in phytopathogenic *Pseudomonas* differs from *P. aeruginosa*

OPS structures of plant‐associated *Pseudomonas* species show a low monosaccharide variability compared to mammalian pathogens (Molinaro *et al.*, 2009). The structural similarity of rhamnose‐rich *P. aeruginosa* CPA and the rhamnose‐rich chemical composition of OPS from other *Pseudomonas* strains suggests a genetic analogy. In our genetic analysis, however, we could not identify a CPA cluster orthologue in most of the investigated *Pseudomonas* genomes. Besides *P. aeruginosa* PA14 and PA7, only *P. fluorescens* Pf0‐1, *P. chlororaphis* ATCC17415 and *P. brassicacearum* NFM421 apparently contain a complete orthologue of the *P. aeruginosa* PAO1 CPA cluster (Lam *et al.*, 2011). Notably, in contrast to *P. aeruginosa* CPA, many of the known OPS structures from plant‐associated *Pseudomonas* strains contain l‐rhamnose, not d‐rhamnose (Molinaro *et al.*, 2009; Zdorovenko and Veremeichenko, 2001; Zdorovenko *et al.*, 2001). Nevertheless, *P. aeruginosa* core‐OS contains an l‐rhamnose residue that is transferred by the rhamnosyltransferases MigA (PA0705) or WapR (PA5000). Possibly, one specific rhamnosyltransferase, potentially orthologous to MigA or WapR, could be responsible for the synthesis of l‐rhamnose‐rich OPS. Alternatively, stereochemical differences in OPS structure could be enabled by OPS synthesis by a different, yet unknown, set of genes.

The search for orthologues of *P. aeruginosa* OSA synthesis proteins yielded hits in all analysed predicted proteomes and uncovered a putative syntenic region of the OSA cluster in the *Pseudomonas* genomes. Compared to the complete cluster in *P. aeruginosa* PAO1 (17 genes), most of these cluster‐orthologues contained only two to three genes. The two genes conserved in all *Pseudomonas* genomes code for an epimerase (WbpM) and a glycosyltransferase (WbpL) that possess a generic function for OPS synthesis in *P. aeruginosa*. However, these very short clusters are unlikely to facilitate synthesis of a complete OPS. The lack of Wzx, Wzy and Wzz/Wzz2, which define the Wzy‐dependent OSA biosynthesis pathway in *P. aeruginosa* PAO1, indicates a Wzy‐independent OPS synthesis in most of the analysed strains (Islam and Lam, 2014; Kalynych *et al.*, 2011). Two investigated *P. syringae* pv. *tomato* OPS‐chains consist of an l‐rhamnose backbone with lateral *N*‐acetyl‐d‐fucosamine (d‐Fuc*N*Ac) residues (Knirel *et al.*, 1993, 1998), whereas *P. cichorii* OPS is composed of alternating *N*‐acetyl‐l‐fucosamine (l‐Fuc*N*Ac) and *N*‐acetyl‐d‐quinovosamine (d‐Qui*N*Ac) (Jimenez‐Barbero *et al.*, 2002) saccharide units. Their generally heteropolymeric structure contradicts a synthesis through an ABC transporter‐dependent pathway or *S. enterica* serovar Borreze‐specific synthase‐dependent pathway. The partially conserved cluster raises questions about the evolution of OPS biosynthesis in *Pseudomonas*. Our results may suggest that in the analysed genomes the majority of the OPS cluster genes were lost or are differently organized. Taken together, additional, as yet unknown, genes, possibly organized in another cluster, could be required for OPS synthesis in these plant‐associated *Pseudomonas* strains.

### WbpL has a conserved function in *Pseudomonas*

In *P. aeruginosa*, the glycosyltransferase WbpL (PA3145) catalyses the initiating step of CPA and OSA biosynthesis. Knockout of *wbpL* leads to synthesis of LPS lacking OPS in *P. aeruginosa* (Bélanger *et al.*, 1999; Rocchetta *et al.*, 1998, 1999) and *P. putida* (Junker *et al.*, 2001). Our analyses indicate that WbpL function is generally conserved in *Pseudomonas* and its knockout results in OPS‐deficient LPS in both *Pst* and *Pci*. While transient complementation of *Pci* ∆*wbpL* confirms WbpL function in OPS synthesis, no plasmid‐driven complementation of OPS synthesis could be achieved in *Pst* ∆*wbpL*. However, transient expression of *Pst‐wbpL* in *Pci* ∆*wbpL* reconstitutes OPS synthesis and demonstrates its conserved function in OPS synthesis. The unsuccessful *Pst* ∆*wbpL* complementation might further indicate an influence of *wbpL* expression level on OPS initiation. Accordingly, we observed an increase of OPS‐deficient LPS species in *Pci* ∆*wbpL + Pci‐wbpL* compared to the wild‐type. Possibly, the initiation frequency decreases due to a changed *wbpL* expression level and leads to the altered substitution of LPS with OPS. Bronner *et al. *(1994) observed a similar gene dosage effect when transiently expressing OPS‐synthesis components in *Escherichia coli* and assumed that increasing the copy number of the ABC‐transporter components relative to the polysaccharide synthesis enzymes leads to a reduction of the average OPS length. Since the precise coordination of the ratio of OPS‐synthesis components appears to be crucial, alterations in *wbpL* expression might disturb OPS synthesis initiation. Thus, ectopic *wbpL* expression in *Pst* ∆*wbpL *mutants might not result in reconstitution of OPS synthesis. It cannot be excluded that polar effects of the *wbpL* knockout on expression of the *wbpK* gene, which is located directly upstream of *wbpL*, lead to the loss of OPS but with regard to WbpK function it seems rather unlikely. It is assumed that WbpK catalyses the conversion of UDP‐4‐keto‐d‐Qui*N*Ac to UDP‐d‐Fuc*N*Ac in *P. aeruginosa*, which is an essential step for the synthesis of d‐Fuc*N*Ac‐containing OPS (King *et al.*, 2009). The OPS of *Pst*, however, contains only non‐stoichiometric d‐Fuc*N*Ac side chains (Knirel *et al.*, 1993, 1998).

### *O*‐polysaccharide‐deficient *Pst* and *Pci* strains are viable *in planta* and cause disease symptoms

To date, only a few reports on the function of OPS during plant infection exist and most mechanistic insights derive from bacteria–host interaction studies in mammals. For most mammalian pathogens, loss of OPS has a negative impact on virulence (Raetz and Whitfield, 2002). Several studies report a diminished symbiosis capability of plant root symbionts (Carlson *et al.*, 2010; Lerouge and Vanderleyden, 2002; Ormeño‐Orrillo *et al.*, 2008) and reduced virulence of plant‐pathogenic bacteria (Berry *et al.*, 2009; Drigues *et al.*, 1985; Li *et al.*, 2014; Petrocelli *et al.*, 2012; Rapicavoli *et al.*, 2018; Schoonejans *et al.*, 1987) that are defective in OPS synthesis. These findings endorse the hypothesis that OPS is also essential for plant‐pathogen virulence. An OPS‐deficient *P. syringae* pv. *syringae* 61 ∆*galU* mutant strain shows reduced viability *in planta* (Deng *et al.*, 2010). GalU is required for synthesis of the monosaccharide precursor UDP‐glucose. Knockout of *galU* leads to OPS‐loss due to truncation of core‐OS in *Pseudomonas* but also affects other pathways, such as exopolysaccharide synthesis and protein glycosylation, which might additionally impair virulence (DeLucia *et al*., 2011; Kocincova and Lam, 2011; Liao *et al.*, 2014; Molinaro *et al.*, 2009). By contrast, a targeted disruption of OPS‐synthesis by knockout of the OPS‐specific glycosyltransferase *wbpL* allows to specifically address the role of OPS in plant–microbe interactions.

Here, we used the well‐established *Pst*‐*A. thaliana* pathosystem and infected lettuce leaves with *Pci* to study the role of OPS during infection processes. Infection experiments with the OPS‐deficient *Pst* ∆*wbpL* mutant revealed a reduced entry into, and slower amplification within, the leaf apoplast, but not a loss of virulence. Indeed, compared to *Pst*‐infected leaves, disease symptoms in *Pst* ∆*wbpL*‐infected leaves develop more slowly but eventually reach similar severity at later time points. *Pci* ∆*wbpL* causes lesions with reduced sizes in wounded lettuce midrib and less severe infection symptoms in lettuce leaves after surface inoculation. Apparently, OPS‐deficient ∆*wbpL* strains can still amplify *in planta* and cause disease symptoms (Fig. 5 and Video S1). Recent reports describe an earlier recognition of OPS‐defective *X. fastidiosa* by plant innate immunity (Rapicavoli *et al.*, 2018). Possibly, the *Pst* ∆*wbpL* might trigger *A. thaliana* immunity earlier than wild‐type bacteria. Therefore, initial amplification of bacteria within the plant might be delayed until the pathogen can overcome defence responses, e.g. with type III‐secreted effectors like AvrPtoB and AvrPto, and eventually cause disease (Macho and Zipfel, 2015; Wei and Collmer, 2018). Additionally, OPS‐deficient mutants could be more sensitive to plant antimicrobial metabolites. OPS may provide a general protection against plant immune recognition and defence responses (Ranf *et al.*, 2015). Spray inoculation shows that the ability to enter host tissues is reduced for *Pst* ∆*wbpL*. Similarly, disease symptoms on lettuce leaves spray‐inoculated with *Pci* ∆*wbpL* are strongly reduced compared to leaves sprayed with wild‐type *Pci*. Hence, reduced motility of the ∆*wbpL* strain might additionally affect its amplification rate and dissemination *in planta*. Taken together, this suggests that the OPS‐defective mutants are not impaired in their ability to cause disease but that the reduced virulence correlates with a reduced capacity to colonize host tissue.

### Loss of the *O*‐polysaccharide influences bacterial motility

Entering of host tissue through stomata requires active movement by the *Pst* cells (Zeng *et al.*, 2010). Here, we show a possible link between reduced bacterial titre in spray‐inoculated plants and impaired motility of *Pst* ∆*wbpL*, as observed in *in vitro* swarming motility assays. Several studies report reduced motility for Gram‐negative bacteria lacking OPS (Huang *et al.*, 2006; Kim and Surette, 2005; Petrocelli *et al.*, 2012). However, it is not clear whether this is due to a motility‐enabling effect of the OPS or a general change of the bacterial cell wall composition and polarity. For example, in *P. aeruginosa* PAO1, a truncated LPS structure leads to a decrease of flagellar motility due to modulation of cell‐surface attachment (Lindhout *et al.*, 2009). Although flagella and LPS synthesis share some precursor synthesis steps, flagellar assembly was not linked with genetic alterations leading to OPS‐deficient LPS. However, attachment to abiotic surfaces was increased and motility was impaired (Lindhout *et al.*, 2009). Furthermore, reduced motility and increased surface attachment seem to be linked to an increased biofilm formation in OPS‐defective bacterial strains (Lee *et al.*, 2010). Bacterial motility is essential for entering the host tissue and for epiphytic fitness (Haefele and Lindow, 1987; Tans‐Kersten *et al.*, 2001; Zeng *et al.*, 2010). We observed that a loss of OPS impairs motility, which in turn may contribute to reduced virulence *in planta*. Future studies of possible alterations in surfactant release or surface composition and polarity will clarify how OPS influences motility during plant–bacteria interactions. The OPS chain length distribution varies significantly between different *Pseudomonas* species, pathovars and strains (Kutschera and Ranf, 2019b). The modulation of OPS chain length might therefore constitute a mechanism for fine‐tuning of cell‐surface polarity to facilitate motility as well as adhesion and biofilm formation in the context of host adaption.

## Experimental Procedures

### Plant growth conditions

*Arabidopsis thaliana* ecotype Col‐0 plants were grown on soil in climate chambers under short day conditions with 8 h of light, 22/18 °C (day/night) and 55% relative humidity.

### Bacterial strains and growth conditions

*Pst*, *Pci* and respective mutants were grown at 26 °C in King’s B (KB) medium liquid culture with shaking (230 rpm) or on KB and low‐salt lysogeny broth (LSLB) agar plates with 2% (w/v) agar. *E. coli* DH5α and S17‐1 were grown at 37 °C in lysogeny broth (LB) liquid culture with shaking (230 rpm) or on LB agar plates with 2% (w/v) agarose.

### Genomic analysis

The annotated *P. aeruginosa* PAO1 genome [GenBank assembly accession: GCA_000006765.1, Stover *et al. *(2000)] was used as reference to identify orthologous LPS biosynthesis genes in other genomes. Reciprocal BLAST experiments of protein‐coding regions were conducted against the proteomes listed in Table S1. A python script was established using BLASTP to identify orthologues of the query sequences in each proteome set up as individual local databases. The sequence of the first hit was retrieved by the algorithm and sequence identity was calculated from the quotient of hit sequence length and corresponding identities. Heatmaps were created from sequence identity values of the BLAST results and dendrograms were calculated from the corresponding Euclidean distances. Scripts are available online (https://gitlab.com/alexander.kutschera/quickblast). Gene synteny was analysed using SyntTax with standard settings (Oberto, 2013).

### Gene knockout in *Pst* and *Pci*

Knockout plasmids were constructed using the pGGKO‐blue plasmid derived from pK18mobsacB (oligonucleotides: Table S2). Flanking sequences (800–1000 bp) up‐ and downstream of the target gene were PCR‐amplified from genomic DNA (oligonucleotides; Table S2) and inserted into the pGGKO‐blue backbone by Golden Gate cloning using *Bpi*I. Precursor knockout plasmids were transformed into *E. coli* DH5α cells and isolated after amplification using standard protocols. The Gm^R^ gene was amplified from plasmid pPS856 (Hoang *et al.*, 1998) and inserted between the flanking sequences (oligonucleotides; Table S2). Knockout plasmids were amplified in *E. coli* DH5α, verified by restriction enzyme digestion and sequencing. Mutants of *Pst* and *Pci* were generated as described (Kvitko and Collmer, 2011). Mutants were verified by PCR and amplicon sequencing (oligonucleotides; Table S2).

### Knockout complementation in *Pst* and *Pci*

Complementation plasmids are based on the backbone of the pGGKO‐blue plasmid. They contain the respective complementing gene under control of the promoter from the Gm^R^ cassette of pPS856 (oligonucleotides; Table S2). Gene sequences were PCR‐amplified from genomic DNA. Together with the promoter, they were inserted into the pGGKO‐blue backbone by Golden Gate cloning using *Bpi*I. Complementation plasmids were amplified in *E. coli* DH5α, verified by restriction analysis and sequencing, and transformed into *E. coli* S17‐1 cells. They were used to conjugate the plasmids to *Pst* or *Pci* via biparental mating as described above.

### LPS extraction and analysis

#### Extraction of crude LPS

Crude LPS was extracted from bacterial cells as described by Hitchcock and Brown (1983). Two millilitres of a KB liquid overnight culture (*A*_600_ of 1.0) was harvested and washed with 0.15 M NaCl solution. The cells were resuspended in 1 mL lysis buffer (2 M Tris‐HCl pH 6.8, 4% (w/v) SDS, 8% (v/v) 2‐mercaptoethanol, 10% (w/v) glycerol, 0.02% (w/v) bromophenol blue in Millipore‐grade water (MP‐water)) and denatured at 100 °C for 5 min. After cooling to RT, 20 μL proteinase K (10 mg/mL, Sigma‐Aldrich, St Louis, MO, USA) was added to the suspension and incubated at 50 °C overnight.

#### Hot phenol‐water and phenol‐chloroform‐petroleum ether extraction of LPS

Preparation, purification and yields of LPS from bacterial cells are specified in the supporting information. Briefly, bacteria were grown in KB medium at 26 °C under shaking. Harvested cells were washed with solvents and enzyme digested. LPS was isolated via hot phenol‐water extraction and in most cases subsequent phenol‐chloroform‐petroleum ether extraction as described by Westphal and Jann (1965) and Galanos *et al. *(1969), respectively.

### Gel permeation chromatography purification of LPS preparations

LPS of *Pst* was further purified by gel permeation chromatography (GPC) on Sephacryl S‐400 HR (GE Healthcare) on a column (2.5 × 120 cm) as described (Jimenez‐Barbero *et al.*, 2002; Kutschera *et al.*, 2019). Purification of 24.3 mg in one run yielded 11.3 mg of purified LPS. Selected LPS preparations were further fractionated on Sephacryl S‐200 HR (GE Healthcare, Chicago, IL, USA) on a column (1.5 × 120 cm) using a desoxycholate (DOC)‐containing buffer as described (Kutschera *et al.*, 2019; Peterson and McGroarty, 1985). The exact procedure and the respective yields are specified in the supporting information.

### Urea SDS‐PAGE, Bis‐Tris NuPAGE and silver staining

Urea SDS‐PAGE or Bis‐Tris NuPAGE and silver staining were performed as described (Kittelberger and Hilbink, 1993) with minor modifications. The detailed procedure is described in the supporting information.

### Mild acidic hydrolysis of LPS

Mild acidic hydrolysis of LPS was performed as described (Ranf *et al.*, 2015) with the following modification: re‐extraction of the water phase was performed four times with CHCl_3_.

### Bacterial infection assay

Bacterial infection experiments with *Pst* and *Pci* strains were performed as described by Zipfel *et al. *(2004) and Starkey and Rahme (2009), respectively. Briefly, for spray inoculation, leaves of 6‐week‐old *A. thaliana* plants or leaves from store‐bought romaine lettuce were inoculated by spraying with a 10^8^ CFU/mL suspension of *Pst* or *Pci* wild‐type and ∆*wbpL* bacteria containing 0.04% Silwet‐L77 (Lehle Seeds) and 10 mM MgCl_2_. For pressure infiltration, suspensions of *Pst* or the *Pst* ∆*wbpL *mutant strain with 10 mM MgCl_2_ were infiltrated into *A. thaliana* leaves with a needleless syringe at 10^6^ CFU/mL if not stated otherwise. Stab inoculation of lettuce midrib with *Pci* strains (10^4^ CFU/mL) was performed as described by Starkey and Rahme (2009). Infected *A. thaliana* leaves were harvested 4 and 72 h after inoculation. Bacterial CFU in the inoculum and leaf samples were determined by counting of colonies’ serial dilution on LSLB plates supplemented with 75 μg/mL rifampicin. Disease symptoms on the lettuce leaves and midrib were monitored over time. The size of the emerging lesions after stab inoculation was measured from images using Fiji (Schindelin *et al.*, 2012).

### Bacteria swarming motility assay

Protocols described by Burch *et al. *(2012) and Tremblay and Déziel (2008) were adapted and modified for the *Pst* and *Pci* swarming motility assessment. Fifty millilitres of freshly prepared KB medium containing 0.4% (w/v) agarose was poured into Petri dishes and dried for 45 min. For complementation strains, 50 μg/mL kanamycin was added to the medium. Bacterial cells were harvested from 3 mL KB overnight cultures by centrifugation (2000 ***g***, RT, 5 min) and resuspended in phosphate‐buffered saline to an OD_600_ of 3.0. 5 μL of the suspension was spotted on the middle of a dried agar plate and dried for 5 min. After sealing, the plates were incubated for 16 h at 26 °C in light, photographed and bacterial motility was digitally assessed using Fiji (Schindelin *et al.*, 2012).

## Supporting information


**Fig. S1** 12% Bis‐Tris NuPAGE gel and silver staining of lipopolysaccharide (LPS) preparations from *Pseudomonas cichorii *ATCC10857/DSM50259 (*Pci*) wild‐type (WT), ∆*wbpL::Gm* *^R ^*(∆*wbpL*) deletion mutant strains complemented with plasmid‐expressed *wbpL *orthologue from either *Pci *(+*Pci‐wbpL*) or *Pst *(+*Pst‐wbpL*). LPS was isolated by hot phenol‐water extraction followed by a phenol‐chloroform‐petroleum ether extraction. The amount of applied LPS is indicated below the respective lane.

**Fig. S2** Exemplary images of (A) lettuce midrib stab infected and (B) lettuce leaves spray inoculated with *Pseudomonas cichorii *ATCC10857/DSM50259 wild‐type (WT) and ∆*wbpL *deletion mutants.

**Table S1** Proteomes used for BLAST experiments.

**Table S2** Oligonucleotides.

**Text S1** Experimental procedure details. Hot phenol‐water and phenol‐chloroform‐petroleum ether extraction of LPS; GPC of LPS preparations using a desoxycholate‐containing buffer (DOC‐GPC), urea SDS‐PAGE, Bis‐Tris NuPAGE and silver staining.

**Video_S1** Time lapse video of *Arabidopsis thaliana *plants infected with *Pseudomonas syringae *pv.* tomato* DC3000 wild‐type (WT) and ∆*wbpL* deletion mutants (∆). Leaves were inoculated by syringe‐infiltration with 10^7^ CFU/mL *Pst* WT or *Pst* ∆*wbpL* suspension. Experiment was repeated two times with 10^6^ CFU/mL bacterial suspension and one time with spray inoculation (10^8^ CFU/mL) with similar results.

## Data Availability

The data that support the findings of this study are available from the corresponding author upon reasonable request.
