# Supplementary material for: Loss of wbpL disrupts O‐polysaccharide synthesis and impairs virulence of plant‐associated Pseudomonas strains
Source: Mol Plant Pathol. 2019 Sep 27;20(11):1535–49. doi: 10.1111/mpp.12864 (PMC6804347; doi:10.1111/mpp.12864)
Supplement: Supplementary file 1 — Fig. S1 12% Bis‐Tris NuPAGE gel and silver staining of lipopolysaccharide (LPS) preparations from Pseudomonas cichorii ATCC10857/DSM50259 (Pci) wild‐type (WT), ∆wbpL::Gm R (∆wbpL) deletion mutant strains complemented with plasmid‐expressed wbpL orthologue from either Pci (+Pci‐wbpL) or Pst (+Pst‐wbpL). LPS was isolated by hot phenol‐water extraction followed by a phenol‐chloroform‐petroleum ether extraction. The amount of applied LPS is indicated below the respective lane. [file MPP-20-1535-s001.pdf]

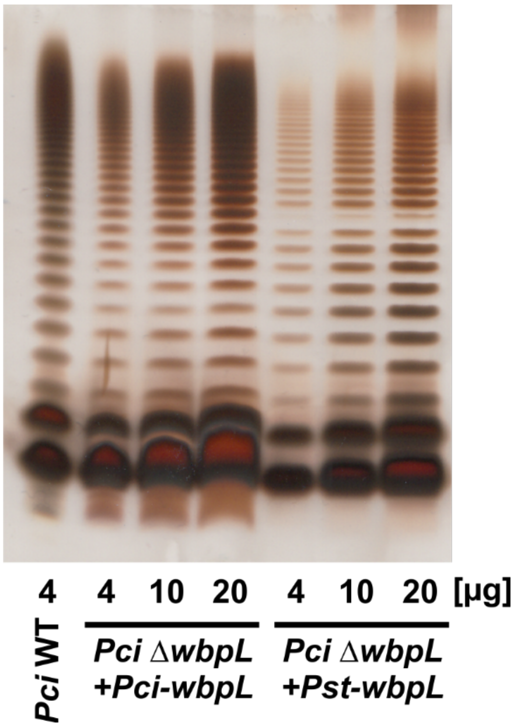

Figure S1: 12% Bis-Tris NuPAGE gel and silver staining of lipopolysaccharide (LPS) preparations from *Pseudomonas cichorii* ATCC10857/DSM50259 (*Pci*) wild-type (WT), Δ*wbpL::Gm*^{R} (Δ*wbpL*) deletion mutant strains complemented with plasmid-expressed *wbpL* ortholog from either *Pci* (+*Pci-wbpL*) or *Pst* (*Pst-wbpL*). LPS was isolated by hot phenol-water extraction followed by a phenol-chloroform-petroleum ether extraction. The amount of applied LPS is indicated below the respective lane.
