# Supplementary material for: Loss of wbpL disrupts O‐polysaccharide synthesis and impairs virulence of plant‐associated Pseudomonas strains
Source: Mol Plant Pathol. 2019 Sep 27;20(11):1535–49. doi: 10.1111/mpp.12864 (PMC6804347; doi:10.1111/mpp.12864)
Supplement: Supplementary file 3 — Table S1 Proteomes used for BLAST experiments. [file MPP-20-1535-s003.docx]

**Table S1:** Proteomes used for BLAST experiments

| **Organism** | **UniProt proteome ID** | **Reference proteome** |
| --- | --- | --- |
| *Pseudomonas aeruginosa* PAO1 | UP000002438 | Yes |
| *Pseudomonas aeruginosa* PA7 | UP000001582 |  |
| *Pseudomonas aeruginosa* PA14 | UP000000653 |  |
| *Pseudomonas brassicacearum* NFM421 | UP000006692 |  |
| *Pseudomonas chloroaphis* ATCC 17415 | UP000281444 |  |
| *Pseudomonas cichorii* JBC1 | UP000019031 | Yes |
| *Pseudomonas protegens* Pf-5 | UP000008540 | Yes |
| *Pseudomonas fluorescens* Pf0-1 | UP000002704 |  |
| *Pseudomonas fuscovaginae* | UP000037931 | Yes |
| *Pseudomonas protegens* Cab57 | UP000031621 |  |
| *Pseudomonas putida* KT2440 | UP000000556 | Yes |
| *Pseudomonas syringae* pv. *actinidiae* ICMP 18807 | UP000015729 |  |
| *Pseudomonas syringae* pv. *japonica* M301072 | UP000004471 |  |
| *Pseudomonas syringae* pv. *maculicola* ES4326 | UP000003811 |  |
| *Pseudomonas syringae* pv. *maculicola* M4a | UP000037879 |  |
| *Pseudomonas syringae* pv. *phaseolicola* 1448A | UP000000551 |  |
| *Pseudomonas syringae* pv. *syringae* B728a | UP000000426 |  |
| *Pseudomonas syringae* pv. *tomato* DC3000 | UP000002515 | Yes |
|  |  |  |
|  |  |  |
|  |  |  |
|  |  |  |

P. fluorescens
