# Supplementary material for: Loss of wbpL disrupts O‐polysaccharide synthesis and impairs virulence of plant‐associated Pseudomonas strains
Source: Mol Plant Pathol. 2019 Sep 27;20(11):1535–49. doi: 10.1111/mpp.12864 (PMC6804347; doi:10.1111/mpp.12864)
Supplement: Supplementary file 4 — Table S2 Oligonucleotides. [file MPP-20-1535-s004.docx]

| **Application** | **Name** | **Nucleotides (5`-> 3`)** |
| --- | --- | --- |
| pGGKO-blue construction | pGGKObb-f | TTTGAAGACTGCTAGCTTCACGCTGC |
|  | pGGKObb-r | TTTGAAGACTCTCGAGTTAATGAATCGGCCAACG |
|  | ggLacZ-f | TTTCTCGAGTGTCTTCGTCACAGCTTGTC |
|  | ggLacZ-r | TTTGCTAGCAGTCTTCGCAGCTGGCAC |
| *Pst* KO plasmid construction | PstA-f | TTTGAAGACTCTCGAGGACAACCGGTGGGAAAGC |
|  | PstA-r | TTTGAAGACGCGGCCGCCTCAATCATGCTTGTCGTG |
|  | PstB-f | TTGAAGACGCGGCCGCTTGATAACGAAGCGGGTTTCTGG |
|  | PstB-r | TTTGAAGACTGCTAGCCACCGTCTTGCCCTTGATG |
| *Pci* KO plasmid construction | PciA-f | TTTCTCGAGCGGTGAACTGGCCGCAATTGC |
|  | PciA-r | TTTGCGGCCGCCATTTTTGCGTTCCAG |
|  | PciB-f | TTTGCGGCCGCTTGATGATTTGAGTATTCGCG |
|  | PciB-r | TTTTGCTAGCCCGTCTGCCCCTTGATGC |
| Gm^R^ insertion | ggGmR-f | TGAAGACTTGGCCAGCTCGAATTGGGGATCTTG |
|  | ggGmR-r | TGAAGACTTGGCCGAGCTCGAATTAGCTTCAAAAGC |
| PCR verification in *Pst* | vPst-f | CAGGTGCGACAGGTTTCGTGG |
|  | vPst-r | GCAGAACGCTGTTCAAGCTCG |
| PCR verification in *Pci* | vPci-f | CCTTCAGCATGGGGCGTACTC |
|  | vPci-r | CTCCAGTATCTGCCGGCACAG |
| Sequencing | Seq-A | GTTACCACCGCTGCGTTCGGTC |
|  | Seq-B | GATATCGACCCAAGTACCGCCAC |
| Gm^R^ promotor | pGmR-f | TGAAGACGTTCGAGTCAACAGCAATGGATC |
|  | pGmR-r | TGAAGACAACATCGTTGCTGCTCCATAACATC |

**Table S2:** Oligonucleotides
