# Supplementary material for: Loss of wbpL disrupts O‐polysaccharide synthesis and impairs virulence of plant‐associated Pseudomonas strains
Source: Mol Plant Pathol. 2019 Sep 27;20(11):1535–49. doi: 10.1111/mpp.12864 (PMC6804347; doi:10.1111/mpp.12864)
Supplement: Supplementary file 5 — Text S1 Experimental procedure details. Hot phenol‐water and phenol‐chloroform‐petroleum ether extraction of LPS; GPC of LPS preparations using a desoxycholate‐containing buffer (DOC‐GPC), urea SDS‐PAGE, Bis‐Tris NuPAGE and silver staining. [file MPP-20-1535-s005.docx]

**Experimental Procedure Details**

1. **Hot phenol-water and phenol-chloroform-petroleum ether extraction of LPS**

*Preparation and purification of LPS of P. syringae pv. tomato DC3000*

Wild-type bacteria were grown in King‘s B medium at 26 °C under constant shaking (230 rpm) to an absorbance of ~1.0 at 600 nm, then harvested by centrifugation (3,000 × g) for 20 min at 4 °C, resuspended in a small volume of MP-water, boiled for 45 min and lyophilized. The bacterial mass was washed with ethanol, acetone (twice) and diethyl ether, and subsequently dried (recovery, 5.11 g). The pellet was resuspended in MP-water (approximately 17 mg/mL), sequentially treated for 24 h at RT with DNase/RNase and proteinase K (100 μL of 1 mg/mL solutions per gram dry weight for each enzyme; note: usually 100 μL of 10 mg/mL solutions are used per gram dry weight), then underwent dialysis (14-kDa cutoff) and lyophilization (1.52 g bacterial mass). For hot phenol-water extraction (PW extraction) (Westphal and Jann, 1965), bacteria were resuspended in 45% aqueous phenol (10 mL per g bacteria) and stirred for 20 min at 68 °C. After centrifugation (5,600 × g) for 20 min at 4 °C, the upper water phase was collected. The extraction was repeated with the same volume of MP-water as had been collected. Combined water phases and the phenolic phase were dialyzed against deionized water at RT (14-kDa cutoff) and lyophilized. Prior to lyophilization, the dialyzed phenolic phase (PP) was centrifuged (600 × g for 5 min at 20 °C) and divided into supernatant (sup) and sediment (sed). LPS recovered from the water phase (0.35 g; Pst WP) was used as such in further experiments. A phenol–chloroform–petroleum ether extraction (Galanos et al., 1969) was performed with the material of the PP (0.18 g PP-sup; 0.61 g PP-sed). However, this did not result in any significant yields of further *Pst* WT LPS.

*Preparation and purification of LPS of P. syringae pv. tomato DC3000 ∆wbpL*

LPS of *P. syringae* pv. *tomato* DC3000 ∆*wbpL* was isolated similarly as described above for the wild-type LPS. PW-extraction of 4.28 g of washed and enzyme-treated (100 μL of 10 mg/mL enzyme solution per gram dry weight) bacterial cells led to the following yields: 76.5 mg WP, 0.49 g PP-sup, 2.91 g PP-sed. PP phases underwent phenol–chloroform–petroleum ether extraction (Galanos et al., 1969). For this, each pool was resuspended with an Ultra-Turrax in phenol (90%)–chloroform–petroleum ether (40–60 °C) (2:5:8 (vol/vol/vol); 50 mg/mL for PP-sup, 100 mg/mL for PP-sed); the respective suspension was subsequently stirred for 30 min at room temperature and centrifuged (6,000 × g) for 20 min at 20 °C. Each supernatant was collected and the extraction was repeated twice, respectively. Combined supernatants were evaporated in vacuum at 60 °C until phenol crystallization had begun (at room temperature and normal pressure). LPS from PP-sup was precipitated over 72 h at 4 °C with 800 μL MP-water and collected by centrifugation (4,400 × g) for 20 min at 20 °C (Pst PP-sup sed). The material of the PP-sed was unusually viscous. Therefore, it was diluted with 6 mL 90% phenol in total. Subsequently, 2.6 mL MP-water was added, LPS was precipitated over 72 h at 4 °C and collected by centrifugation (4,400 × g) for 20 min at 20 °C (Pst PP-sed sed). The precipitates were washed twice with 80% phenol (centrifugation at 4,400 × g for 20 min at 20 °C) and three times with acetone (centrifugation at 4,400 × g for 20 min at 4 °C) and dried afterwards. This resulted in the following yields: Pst PP-sup PCPI-sup water-precipitate: 0.103 g (this LPS was used in further experiments); Pst PP-sed PCPI-sup water-precipitate: 1.03 g.

*Preparation and purification of LPS of P. syringae pv. tomato DC3000 ∆wbpL complemented with Pst-wbpL*

LPS of *P. syringae* pv. *tomato* DC3000 ∆*wbpL* complemented with *Pst-wbpL* was isolated similarly as described above for the ∆*wbpL* LPS. PW-extraction of 1.16 g of washed and enzyme-treated bacterial cells led to the following yields (for sufficient phase separation, centrifugation had to be performed at 8,000 × g): 0.12 g WP (contained no LPS as judged by NuPAGE), 0.22 g PP-sup, 0.48 g PP-sed. PP phases underwent phenol–chloroform–petroleum ether extraction as described above for isolation of LPS from the ∆*wbpL* strain (resuspension 25 mg/mL for PP-sup, 50 mg/mL for PP-sed). This resulted in the following yields: Pst PP-sup PCPI-sup water-precipitate: 30.9 mg; Pst PP-sed PCPI-sup water-precipitate: 40.8 mg (this LPS was used in further experiments).

*Preparation and purification of LPS of P. cichorii ATCC10857/DSM50259*

Wild-type bacteria were grown in King‘s B medium at 26 °C under constant shaking (230 rpm) to an absorbance of ~1.0 at 600 nm, then harvested by centrifugation (3,000 × g) for 20 min at 4 °C, resuspended in a small volume of MP-water, boiled for 45 min and lyophilized. The bacterial mass was washed with ethanol, acetone (twice) and diethyl ether, and subsequently dried (recovery, 13.5 g). For hot phenol-water extraction (PW extraction) (Westphal and Jann, 1965), bacteria were resuspended in 45% aqueous phenol (10 mL per g bacteria) with an Ultra-Turrax and stirred for 20 min at 68 °C. After centrifugation (5,600 × g) for 20 min at 4 °C, the upper water phase was collected. The extraction was repeated with the same volume of MP-water. The separately treated water phases (WP) and the phenolic phase (PP) were extensively dialyzed against deionized water at 4 °C (14-kDa cutoff) and lyophilized. Before lyophilization, the dialyzed phenolic phase (PP) was centrifuged (600 × g for 5 min at 20 °C) and divided into supernatant and sediment. All pellets were resuspended in water (10 mg/mL) and sequentially treated with DNase/RNase and proteinase K (100 μL of 10 mg/mL enzyme solution per gram of dry weight), then underwent dialysis and lyophilization. This resulted in lyophilizates of 0.23 g and 0.38 g for the water phases respectively, and 0.59 g for the supernatant of the PP (*Pci* WT LPS), which was the LPS containing fraction.

*Preparation and purification of LPS of P. cichorii ATCC10857/DSM50259 ∆wbpL*

LPS from *P. cichorii* ATCC10857/DSM50259 ∆*wbpL* was isolated similarly as described above for LPS of *Pst* ∆*wbpL*. PW-extraction of 3.64 g of washed and enzyme-treated (100 μL of 10 mg/mL enzyme solutions per gram dry weight) bacterial cells led to the following yields: 0.119 g WP, 0.66 g PP-sup, 1.84 g PP-sed. The LPS-containing PP phases underwent phenol–chloroform–petroleum ether extraction, as described above (resuspension 50 mg/mL). This resulted in the following yields: Pst PP-sup PCPI-sup water-precipitate: 264 mg (this LPS was used in further experiments); Pst PP-sed PCPI-sup water-precipitate: 86.1 mg.

*Preparation and purification of LPS of P. cichorii ATCC10857/DSM50259 ∆wbpL complemented with Pci-wbpL*

LPS from *P. cichorii*ATCC10857/DSM50259 ∆*wbpL* complemented with *Pci-wbpL* was isolated similarly as described above for LPS of *Pst* ∆*wbpL*. PW-extraction of 4.47 g of washed and enzyme-treated bacterial cells resulted in a lyophilizate of 0.36 g for the water phase and 1.16 g for the supernatant of the PP. The latter was the major LPS (*Pci* ∆*wbpL*+*Pci-wbpL* LPS)-containing fraction and this LPS was used as such in further experiments.

*Preparation and purification of LPS of P. cichorii ATCC10857/DSM50259 ∆wbpL complemented with Pst-wbpL*

LPS from *P. cichorii* ATCC10857/DSM50259 ∆*wbpL* complemented with *Pst-wbpL* was isolated similarly as described above for LPS of *Pst* ∆*wbpL*. However, in the first precipitation step of the phenol–chloroform–petroleum ether extraction, ethanol was added (1:11 MP-water/ethanol ratio). PW-extraction (yield: 20 mg for the water phase) and subsequent phenol–chloroform–petroleum ether extraction of the resulting PP-phases starting from 1.8 g of washed and enzyme-treated bacterial cells resulted in final yields of 1.6 mg for PP-sup and 6.6 mg for PP-sed. The majority of the S-form LPS was present in PP-sup, therefore this pool was used in further experiments.

1. **GPC of LPS preparations using a desoxycholate-containing buffer (DOC-GPC)**

Selected LPS preparations were further fractionated on Sephacryl S-200 HR (GE Healthcare) on a column (1.5 × 120 cm) using a desoxycholate (DOC) containing buffer as described (Peterson and McGroarty, 1985). Approximately 5 mg LPS were resuspended in 1.5 mL eluting buffer and applied per run to the column. S400-purified *Pst* WT LPS: A representative chromatogram is depicted in Fig. 5A. Resulting sediments were washed four times with 30 mL ethanol (3,363 × g (swing out rotor) for 10 min at 4 °C) and supernatants were discarded. Sediments were dried under a stream of nitrogen, dissolved step wise in 9 mL MP-water, transferred into dialysis tubes (12- to 16-kDa cutoff), and 1 mL of 40 mM MgSO_4_-solution was added. Dialysis against MP-water was performed for three days at 4 °C with twelve water exchanges in total. Final yields: *Pst* WT S200 (DOC) pool 1, 0.649 mg; pool 2, 2.57 mg; pool 3, 1.79 mg; pool 4, 0.249 mg. *Pst* ∆*wbpL* LPS: A representative chromatogram is depicted in Fig. 5B. *Pst* ∆*wbpL* complemented with *Pst-wbpL* LPS: A representative chromatogram is depicted in Fig. 5C. Material of two runs was combined in pools as indicated, lyophilized and further handled as described above. Final yields: *Pst* ∆*wbpL*+*Pst-wbpL* S200 (DOC) pool 1, 0.646 mg; pool 2, 1.054 mg; pool 3, 0.346 mg; pool 4, 6.96 mg; pool 5, 0.321 mg.

1. **Urea SDS-PAGE, Bis-Tris NuPAGE and silver staining**

Sample buffer (aqueous solution containing 2% (w/v) SDS, 5% (v/v) 2-Mercaptoethanol, 20% (w/v) glycerol, 1% (v/v) 0.5 M EDTA pH 8.0 solution and 12.5% (v/v) 0.5 M Tris/HCl pH 6.8 solution) was added to LPS samples. These were heated to 100 °C for 5 min and subsequently separated in a 4% stacking gel and an 18% resolving gel containing 24% urea (w/v) at a current of 90 V using standard PAGE buffer. To make the LPS visible in the gel, it was first incubated in fixing solution (aqueous solution containing 25% (v/v) isopropanol and 7% (v/v) acetic acid) for 2 h and oxidized in fixing solution containing 0.7% (w/v) periodic acid for 5 min. After 5 washing steps with MP-water, the gel was incubated in alkaline silver nitrate solution (aqueous solution containing 20 mM NaOH, 0.3% (v/v) NH_3_ and 0.7% (w/v) AgNO_3_) for 10 min. The gels were washed five times in MP-water and developed in an aqueous solution containing 3% (w/v) Na_2_CO_3_ and 0.02% (v/v) formaldehyde. Development was stopped with 0.05 M EDTA solution after sufficient staining had been achieved. The stained gels were washed three times in MP-water and subsequently scanned. 12% Bis-Tris NuPAGE gels (Thermo Fisher Scientific) were used according to the manufacturer’s instruction. For silver staining, these gels were incubated for 2 h in fixing solution (30% (v/v) ethanol, 10% (v/v) acetic acid in MP-water), oxidized for 10 min in fixing solution containing 0.7% (w/v) periodic acid, and washed three times with MP-water. The gel was stained for 30 min in 0.1% (w/v) aqueous silver nitrate solution (in the dark) and after transferring the gel for 10 seconds into MP-water it was developed in a 3% (w/v) sodium carbonate/0.02% (v/v) formaldehyde solution for approximately 10 min (in the dark). Reaction was stopped with 1% (v/v) acetic acid and the gel was finally washed three times for 10 min with MP-water and subsequently scanned.
